# Mechanoregulation of Bone Remodeling and Healing as Inspiration for Self-Repair in Materials

**DOI:** 10.3390/biomimetics4030046

**Published:** 2019-07-09

**Authors:** Richard Weinkamer, Christoph Eberl, Peter Fratzl

**Affiliations:** 1 Department of Biomaterials, Max Planck Institute of Colloids and Interfaces, 14476 Potsdam, Germany; 2 Fraunhofer Institute for Mechanics of Materials, 79108 Freiburg, Germany; 3 Institute of Microsystems Technology, University of Freiburg, 79110 Freiburg, Germany

**Keywords:** bone remodeling, bone healing, mechanoregulation, mechanobiology, mechanical stimulus, feedback loop, adaptive material, programmable material, control function

## Abstract

The material bone has attracted the attention of material scientists due to its fracture resistance and ability to self-repair. A mechanoregulated exchange of damaged bone using newly synthesized material avoids the accumulation of fatigue damage. This remodeling process is also the basis for structural adaptation to common loading conditions, thereby reducing the probability of material failure. In the case of fracture, an initial step of tissue formation is followed by a mechanobiological controlled restoration of the pre-fracture state. The present perspective focuses on these mechanobiological aspects of bone remodeling and healing. Specifically, the role of the control function is considered, which describes mechanoregulation as a link between mechanical stimulation and the local response of the material through changes in structure or material properties. Mechanical forces propagate over large distances leading to a complex non-local feedback between mechanical stimulation and material response. To better understand such phenomena, computer models are often employed. As expected from control theory, negative and positive feedback loops lead to entirely different time evolutions, corresponding to stable and unstable states of the material system. After some background information about bone remodeling and healing, we describe a few representative models, the corresponding control functions, and their consequences. The results are then discussed with respect to the potential design of synthetic materials with specific self-repair properties.

## 1. Introduction

Bone is an archetypical example for an organ that completely regenerates through a healing process [1,2,3] and serves as a role model in materials engineering [4]. The healing process is most generally associated with the reconnection of separate pieces after bone fracture. The specificity of bone healing in our body is its regenerative character which enables it to return to the pre-fractured state without leaving a scar tissue. This is possible because in an early phase of the healing process cells migrate into the tissue, providing a connection between bone ends. Subsequently, these cells differentiate and form new tissue in response to mechanical stimulation. On a more mesoscopic length scale, bone constantly undergoes a remodeling process. The purpose of this permanent resorption of old material and formation of new material is not only to replace damaged tissue but to also allow for structural adaptation to the most common loading conditions, thereby avoiding local concentration of loads that might induce failure [5,6,7].

This perspective focuses on bone healing and remodeling. Both are described as mechanobiological processes [8,9], i.e., mechanical stimuli that act as key regulators. The effect of mechanical stimulation can be (local) changes of the structure or of material properties. Mechanical forces propagate through materials and are thus able to transmit information. In this way, a defect occurring in a certain position of the material can be “felt” at some distance due to changes in force distributions. This non-local nature of mechanical forces is therefore an effective way of triggering a mechanobiological response of the material at a location different from the actual stimulus, leading to an adaptation of the properties. In the case of bone healing, a cascade of local events is set into motion that can eventually result in the macroscopic structural change of a reunion of two bone fragments.

**Figure 1:**
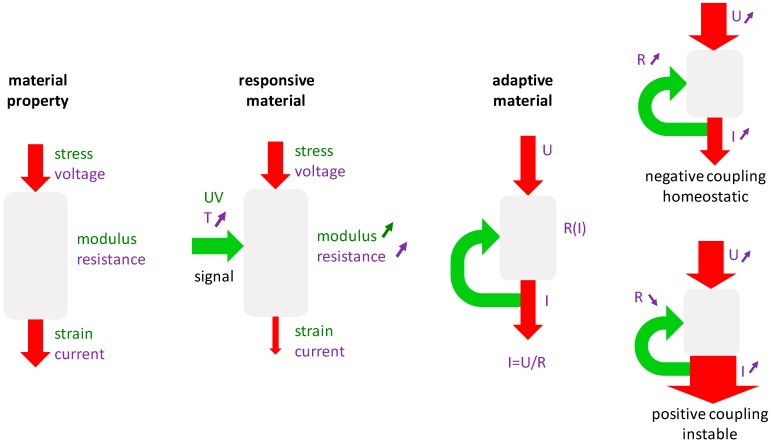
Comparison between different material behaviors: in the simplest case a material property like resistance or modulus is characterized by a fixed value (**left**); in responsive materials (**middle**) the property can be influenced by an external stimulation; in adaptive materials the stimulation is created internally by feeding back a signal related to the material’s output (**right**).

From a theoretical point of view, the crucial material characteristic is the material’s response to a mechanical stimulation. This characteristic is of higher complexity than typical material properties. Figure 1 exemplifies this by analogy to behaviors from well-known simple systems. Generally, a material parameter describes the (linear) relation between cause and effect, like how electrical conductivity (i.e., the inverse of the electrical resistance) describes the resulting current after the application of a specific voltage (Figure 1, left). Similarly, the compliance of a material is a measure of the relative length change of a material in response to an applied compressive load.

A first step from a static to a more dynamic description of material behavior is to consider possible influences of environmental conditions. Taking the example of electrical conductance, the material can respond to a change in temperature by an increase of the resistance. In this way, the material can become a responsive material, since the temperature can be used to influence the resulting current (Figure 1, middle). This form of control is similar to the function of a gate in a transistor. A further step towards more complex material behavior is obtained when the loop is closed so that the controlling signal is not coming from outside but is intrinsically generated. In the Figure 1, right, the resulting current is used to control the temperature, which influences the resistance of the material and in turn affects the current. Thus, again, the control loop is closed. Such feedback loops are characteristic for adaptive systems. It is important to distinguish between negative and positive feedback loops. In a negative feedback loop (or negative coupling), a large outcome (i.e., current in our example) would increase the resistance in the material and, consequently, lower the outcome. This kind of regulation is known for thermostats that regulate the temperature near a desired setpoint. Analogously, the system attains a state of dynamic equilibrium, which is due to the self-regulation of the system typically termed homeostasis in biological systems. For a positive feedback loop (or positive coupling) a large current in our example would induce a reduction of the resistance and, consequently, further increase the resulting current. In this case, the system is unstable and tends to spiral out of control. Classical outcomes are that the material’s response is limited by some boundary conditions, like a lower boundary of the resistance that cannot be crossed; therefore, the system stabilizes by attaining minimum/maximum values. Further, the escalation of the process has severe side effects that put an end to the usual function of the material.

Bone remodeling and healing provide examples of mechanoregulation via negative and positive feedback loops, respectively. In theoretical models of these processes, the mechanoregulation is implemented by functions that link a local mechanical stimulus to a local change in the structure or properties of bone material. These so-called control functions are typically motivated from biology and challenging to measure experimentally. Consequently, computer models play an essential role in the exploration of such systems. In computer models the following two steps are run through iteratively: Firstly, a mechanical assessment is performed under external loading conditions considering the current mechanical properties of the material. The obtained local strains and stresses in the material are then used to calculate the mechanical stimulus. Secondly, the control function converts the stimulus into local changes of structural or material properties, which are consequently updated. With the so changed properties of the material, a new iteration starts.

Due to the biomedical importance of bone healing and remodeling, the mechanobiological aspect of these processes have been studied extensively using computer simulations. The scientific literature provides excellent reviews about computational work addressing the mechanobiological aspects of bone [8] and specifically for bone remodeling [10,11,12] and bone healing [13,14,15,16]. Instead of providing a further review, this perspective discusses a few representative examples of control functions for bone remodeling and healing. The definition of these control functions is sometimes “buried” in the Materials and Methods sections of the corresponding papers and are often described in biological terms without specifying their role as a control function in the sense introduced above. It is the aim of the present perspective to highlight the role of the control function and to provide some intuitive understanding of how choices of the control function influence the resulting repair process.

The paper is organized into two parts. The first part deals with bone remodeling and the second with bone healing. In each part, the biological background of the process is given first. This description prescinds from all molecular and cell biological details and should only provide the background from a materials science viewpoint to understand the model approaches. After some theoretical considerations about the processes, the computer models and their used control functions are presented. The perspective concludes by discussing mechanoregulation in the context of the design of synthetic materials with self-repair properties.

## 2. Bone Remodeling

### 2.1. Biological Background

The processes of bone modeling and remodeling are thought to reduce the fracture risk of bone. Remodeling refers to a renewal of the material by exchanging old, damaged bone with new bone. The aim of remodeling is, therefore, the maintenance of bone. Modeling refers to adding or removal of bone as response to mechanical needs. Here, the aim is an adaptation of the bone structure to a change in the mechanical environment [5,6]. For example, after starting to play tennis, the bones in the racket arm increase their cross-sectional area, while the bone in the opposite arm remains structurally unchanged [17]. The distinction between modeling and remodeling makes sense conceptually. However, both processes are performed by the same bone cells. Osteoclasts are resorbing bone and osteoblasts are forming bone. On the level of the bone cells, remodeling has been defined by a resorption event followed by a formation event. In this case, osteoclasts and osteoblasts work as a “team”, where this team character is highlighted by describing the actuating cells as a bone multicellular unit (BMU). A resorption event that is not followed by formation or, vice versa, a formation event that is not preceded by resorption is then conceived as modeling [5]. It is straightforward to think about processes, which carry both characteristics of modeling and remodeling. In aging people, a resorption by osteoclasts is followed by bone formation, but often the amount of formed bone is less than the resorbed bone. This so-called remodeling imbalance results in age-related loss of bone mass [18]. As a consequence, a standard therapy against age-related bone loss and osteoporosis is intended to slow down the rate of bone turnover by administering bisphosphonates (i.e., anti-resorptive therapy) [19]. A pharmaceutical shutdown of a repair mechanism is an ambivalent response to an increased bone fracture risk in older people. In this perspective, we follow the usual convention to refer with bone remodeling to both remodeling and modeling processes.

Osteoblasts and osteoclasts can form and resorb bone only at free bone surfaces. The foamy structure of trabecular bone provides ample surface and easy access for bone cells. In the dense cortical bone, the surface has to be created by the cells themselves [20]. Osteoclasts dig a roughly circular tunnel with a diameter of approximately 300 µm into the bone and, in their wake, osteoblasts are filling this tunnel with new bone material. In this way, a cylindrically shaped osteon is formed. Remodeling is a rather slow and local process. Trabecular bone resorption can take weeks and formation can even take months [21]. Although the exchanged volume in a remodeling event is rather small, the large number of remodeling events running in parallel leads in trabecular bone to a remodeled volume of 20% of the total volume each year [7].

To allow for an adaptation of the bone structure to changes in the mechanical loading, a mechanosensor has to have a controlling role in bone remodeling. Although the sensing mechanism is still debated, there is general agreement that osteocytes play a key role in bone mechanosensation [22,23]. Osteocytes are differentiated osteoblasts and are the most abundant bone cells. They live inside the mineralized bone matrix and connect with their multiple cell processes to other cells to form a cell network. This cell network is housed in a fluid-filled porous network. A prominent theory of bone-mechanosensation is that mechanical loading on the bones induces a fluid flow through the porous network causing shear forces detected by osteocytes [24,25,26]. Alternatively, it has been proposed that microdamage itself is the trigger of bone remodeling by disrupting some of the cell processes [27]. This disruption would lead to the death of osteocytes and the missing signal of the osteocyte would bring osteoclasts and osteoblasts to the scene [28].

### 2.2. Theoretical and Experimental Results about the Mechanocontrol of Bone Remodeling

**Figure 2:**
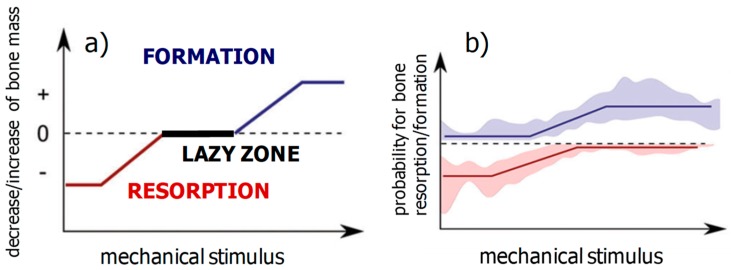
Comparison between a control function, which has been suggested for the mechanoregulation of total bone mass by Harold Frost in his mechanostat theory (**a**), and a control function obtained in experiments on adult mice using a combination of in vivo microcomputed tomography, in vivo loading and Finite Element modeling (**b**) (from [36]).

First, ideas about structural adaption of bone and the mechanoregulation of bone remodeling date back to the late 19th century, with the work of Julius Wolff and Wilhelm Roux. A modern formulation of what is known as Wolff’s law, which governs bone remodeling, reads: bone is locally deposited wherever mechanically needed and is resorbed where it is not needed. Harold Frost described the adaptation of the total bone mass to changes in the mechanical loading using a mechanical feedback model. In his mechanostat model, he introduced an upper threshold of the mechanical stimulation above which the bone mass starts to increase and a lower threshold corresponding to mechanical disuse below which the bone reacts by reducing its mass. Between these two thresholds, a “lazy zone” describes an equilibrium situation with an unchanged bone mass within a range of intermediate mechanical stimulation [29]. A schematic representation of the control function regulating bone resorption and formation in Frost’s mechanostat model is shown in Figure 2a. Using feedback theory, the effect of changes in the setpoint of the mechanostat, e.g., due to hormonal changes, were studied [30]. In a cell-based model of the mechanostat, it has been proposed that the creation of new osteocytes during bone remodeling would provide the possibility to redefine the setpoint of the mechanostat [31]. An unresolved research question is which mechanical quantity is most appropriate to describe the triggering of bone remodeling. Different mechanical stimuli have been proposed, all of which are dynamic and typically related to strain rate [5]. For reasons of convenience, a scalar quantity, e.g., principal strain or strain energy density, is often used in models. Moreover, a static description due to a straight-forward mathematical equivalence between static and dynamic mechanical stimuli in the case of cyclic loading is used [32].

Recently, an important experimental breakthrough was achieved, leading toward a more quantitative description of Wolff’s law. This has been made possible by using in vivo microcomputed tomography (µCT) on small rodents like mice to monitor the progress of bone remodeling [33]. Several three-dimensional images of bone structure in living animals was taken with a time lapse for a few days, which allowed for the sites of bone formation and resorption on the bone surface to localize [34]. In this time period, the imaged bone was mechanically stimulated under controlled conditions and the local mechanical loading on the bone surfaces was calculated using Finite Element (FE) modeling [35,36]. Spatially correlating the results from the in vivo µCT and FE modeling allowed us to deduce the control function (or remodeling rule), i.e., the probability for the occurrence of bone formation or resorption as a function of the strength of the mechanical stimulus (Figure 2b) [35,36]. Several features are remarkable in the measured remodeling rule. (i) Both formation and resorption are mechanically regulated, i.e., the respective curves are increasing for formation and decreasing for resorption. (ii) In the case of bone formation, the mechanoregulation can be well described by a transition between a low probability value for weak mechanical stimulation and a high value for strong stimulation. For resorption, the behavior is mirrored with a drop between two probability values. (iii) The transition between the two values is not sharp, but rather smeared out, i.e., there is no simple threshold above which the formation kicks in or resorption shuts down. Investigation of mice of different age showed that this transition loses further on sharpness with age [36].

### 2.3. Computational Models of the Mechanoregulation of Remodeling

#### 2.3.1. Mechanoregulated Formation and Resorption of Bone Material

The description of the mechanoregulation of bone remodeling using remodeling rules for bone formation and resorption (Figure 2b) allows a straightforward implementation of a remodeling algorithm in a computer model. In the model, the structure of trabecular bone is discretized using a cubic lattice, wherein the binary image white voxels correspond to bone and black to non-bone (i.e., bone marrow). Under a loading usually defined by the daily loads on the specific bone, the stresses and strains in the bone and the mechanical stimulus as a derived scalar quantity can be calculated. With the mechanical stimulus known in each voxel of the bone structure, the remodeling rule is then applied to control possible changes in the structure. A classical implementation in a computer algorithm is used to randomly choose a voxel at the surface. If the voxel is white and corresponds to bone, the remodeling rule is used to obtain the probability for resorbing this bone voxel. Alternatively, if the voxel is black and corresponds to marrow, the remodeling rule provides the probability that this voxel is turned to a bone voxel [37,38]. For the calculation of the mechanical stimulus, it has to be defined whether only the nearest neighbor voxels or a larger environment of the potential site of bone formation contributes [39].

Application of the computer model showed a natural coarsening of the trabecular architecture with age, i.e., the number of trabeculae decreased with the remaining trabeculae becoming thicker so that the bone volume stayed virtually constant [37]. Testing different remodeling rules resulted for all remodeling rules in a trabecular architecture with trabeculae aligned to the external tri-axial loading directions. Differences in the mechanoregulation, however, caused different responses when classical therapies were applied to the virtual bone, i.e., increased mechanical loading due to physical exercise or a decrease in resorption due to an anti-resorptive therapy. Using the computer model, a remodeling rule based on Frost’s mechanostat including a lazy zone (Figure 2a) was found to be in disagreement with experimental findings [38]. Experiments on small animals and humans confirmed that a mechanoregulation including a lazy zone does not provide an adequate description for the mechanical control of bone remodeling [35,36,40].

#### 2.3.2. Mechanoregulated Effective Stiffening/Softening of the Bone Material

In contrast to the model just presented, where the adding or removal of bone form the surface resulted indirectly in a local stiffening or softening of the material on a larger length scale, the model proposed by Rik Huiskes et al. describes bone remodeling more explicitly as a change in local mechanical properties. Again, the bone structure is mapped on a cubic lattice. However, the variable describing the structure is not a binary variable (i.e., bone or non-bone), but a relative bone density m, which is a continuous variable taking values between 0 and 1. The differential equation describing the adaptation of m at the position x at time t is defined as [41]:

(1) $$\frac{dm\left(x,t\right)}{dt}=\{\begin{array}{cc} \tau \left[P\left(x,t\right)-k_{tr}\right]-r_{OC} & \mathrm{for}\;P\left(x,t\right)>k_{tr}\\ -r_{OC} & \mathrm{for}\;P\left(x,t\right)\leq k_{tr} \end{array}$$

where $r_{OC}$ is the relative amount of resorbed bone, P(x,t) is the local stimulus for bone formation, $k_{tr}$ the threshold value for formation, and τ a proportionality constant. The local relative bone density m is then related to the local stiffness E of the material by a Gibson-Ashby type of equation [42],

(2) $$E\left(x,t\right)=C\;m{\left(x,t\right)}^{\gamma }$$

where typically an exponent γ=3 was chosen [43]. This second equation can be used to substitute m in the differential equation by E, resulting in a differential equation for the local stiffness of the material. Consequently, this model approach describes bone remodeling as an adaptive stiffening or softening of the material. Solving this equation under a predefined loading condition results in a homeostatic configuration, which is in equilibrium with the applied load [41].

**Figure 3:**
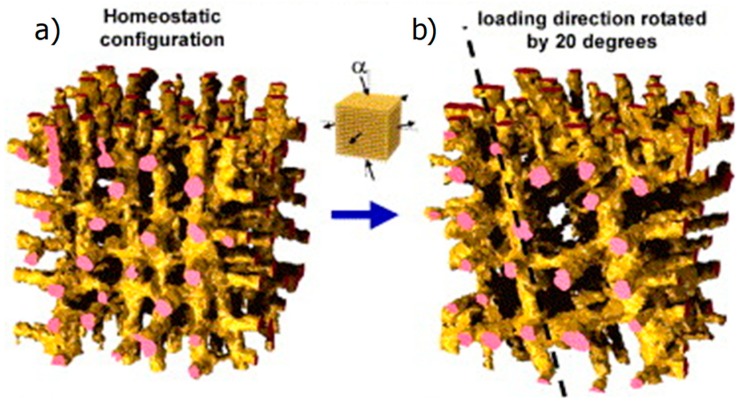
(**a**) The negative feedback loop used as a control function resulted in a homeostatic configuration of the trabecular bone orientation. (**b**) Changing the loading direction to be 20 degrees away from the vertical direction leads to a reorientation of the trabecular architecture (from [45] with permission).

First, implementations of this model in two-dimensions resulted in bone structures with a checkerboard architecture—i.e., a pixel with a bone density close to the maximum value 1 had neighboring pixels with a density of approximately the minimum value of 0 and vice versa [44]. This instability of the model was “tamed” when considering the stimulus of bone formation, P, not only in the mechanical stimulation in the closest vicinity of the site of potential bone formation but also in contributions from a more extended volume. The different contributions where weighted by a spatial influence function, which decays exponentially with the distance from the site at the bone surface [43]. An important result obtained with this model approach was that it explained both the emergence and maintenance of the trabecular architecture. Further, it explained the adaptation to changes in the external loading [41]. Figure 3 shows an example in which first the virtual bone was loaded in three perpendicular directions and the model equations were solved in time until a homeostatic configuration was attained (Figure 3a). Then, the external loading was altered by changing the vertical loading to a loading direction with an angle of α=20° to the vertical direction. The trabecular architecture clearly adapted to this new loading conditions by reorienting its trabeculae along the new loading direction. This reorientation is a rather slow process to that even after a simulation time corresponding to 12 years the reorientation was not completed (Figure 3b) [45].

#### 2.3.3. Bone Remodeling as Damage Management

Bone remodeling can be viewed from the perspective of damage management [27]. The permanent mechanical loading of our bones results in fatigue damage, which accumulates over time. Bone remodeling is counteracting this accumulation of damage and prevents the prevalence of damage production with a stress fracture as its consequence.

The key parameter in the model proposed by Bruce Martin is now the fatigue damage defined as observable crack length per unit area [46]. Two differential equations describe the rate of damage formation and the rate of damage repair. This later rate is proportional to the existing damage and to a so-called damage repair specificity factor, which takes into account that remodeling does not occur spatially random but is directed towards sites of damage accumulation. Based on animal experiments that demonstrated a spatial correlation between sites of damage and sites of remodeling [47], the value of the damage repair specificity factor was estimated to be about 5. The control function in the model describes now the relation between the damage, which is locally present, and the activation frequency, i.e., the probability that within a given time period a new remodeling cycle will be initiated at this local region. In the model, a sigmoidal relationship was chosen for this control function [46].

The model was used to study the interrelation between time scales, specifically of how the time period needed a remodeling cycle that related to the half-life of a local concentration of damage. Importantly, the computational work spotlighted an instability in the repair mechanism. Since bone remodeling starts with bone resorption, the result is an increase in bone porosity and this reduction of material produces higher strains in the remaining bone. However, higher strains are related with an increased damage formation rate that initiates more remodeling events, which first remove the damaged bone. This spiraling out of control of the repair mechanism leads as a final consequence to a stress fracture of the bone [46]. This weakening aspect of bone remodeling has been brought up to explain stress fractures in young military recruits [48].

## 3. Bone Healing

### 3.1. Biological Background

While bone remodeling was about the prevention of fracture by replacing damaged material, bone healing is about the reaction of bone after a fracture occurred and the reunion of broken pieces. Despite all the biological complexity of the healing process [1,2,3,49], specific aspects of bone healing can be described as a mechanobiological process [8]. Different to remodeling, the mechanical stimulus does not influence cell action, but instead influences cell differentiation, i.e., which cells are formed from stem cells/progenitor cells in the first place.

The healing process is classically subdivided into three overlapping phases [50]. During the initial inflammatory phase [51], the fracture zone is cleaned from dead material, activities that restore the blood supply are undertaken, and mesenchymal stem cells congregate. In the repair phase, additional tissue in the form of a fracture callus is formed. With time, this callus turns from a soft callus to a hard callus eventually made of bone. In the final remodeling phase, the superfluous bone material is resorbed, leaving behind an intact healed bone. This rough description of healing highlights two peculiarities. Firstly, that much more material is temporarily formed as needed in the end, so that the last step consists in a removal of this dispensable material. Secondly, that transiently tissues different from bone are present: fibrous tissue, cartilage, and fibrocartilage.

### 3.2. Theoretical Considerations about the Mechanocontrol in Bone Healing

Friedrich Pauwels pioneered mechanobiological thinking in the context of bone healing. Starting from the different mechanical performance of tissues formed during healing, he hypothesized that exactly these tissues are formed in the fracture callus that best perform the mechanical task at hand (i.e., cartilage would form under conditions of hydrostatic pressure since cartilage well resists volume changes). In contrast, fibrous tissues are more suited to resist shape changes and therefore would form under shear stresses [52]. Even closer to an “algorithmic understanding” of bone healing is the Interfragementary Strain Theory by Perren and Cordey [53]. This theory states that only tissues that can withstand the strain in the gap without failure can be formed in the fracture gap. Therefore, the fracture gap should initially be filled with a tough, but generally soft tissue. A consecutive stiffening of this tissue lowers the strain in the callus and stiffer tissues with decreased strain tolerance can be formed. Consequently, a feedback occurs between a stiffening of the callus and a lowering of the strain. The outcome of this positive feedback loop is a hard callus made of bone, which undergoes remodeling during the final phase of healing.

### 3.3. Models of the Mechanoregulation of Bone Healing

#### 3.3.1. Mechanoregulated Models of Tissue Differentiation During Bone Healing

Only the essence of the computer models should be described here, avoiding all the technical details. The starting point of mechanobiological simulations of fracture healing is often the situation after the initial inflammation phase and the disappearance of the hematoma. At this stage, the fracture callus consists of soft tissue and is loaded via the broken bone ends. Each voxel of tissue within the callus is then characterized by three variables: (i) the local loading conditions characterized by the mechanical stimulus; (ii) the tissue type that is present in the voxel (here, some models allow a mixture of different tissues) [54,55]; and (iii) a local “biological potential” [56] that takes into account how healing cannot proceed if some basic biological requirements are not fulfilled, in particular the presence of stem cells and important signaling molecules, as well as a sufficient vascularization. This biological part is usually modeled as a diffusion process. For example, in bone remodeling, a lot of computational work was done to define an adequate mechanical stimulus. Following the ideas of Pauwels, two stimuli corresponding to hydrostatic or shear stresses were proposed [57]: shear stresses and fluid flow [54]. Healing outcomes based on different assumptions were compared [55]. However, a single mechanical stimulus like the volumetric strain proved to be sufficient to describe the healing process under standard loading conditions [56].

**Figure 4:**
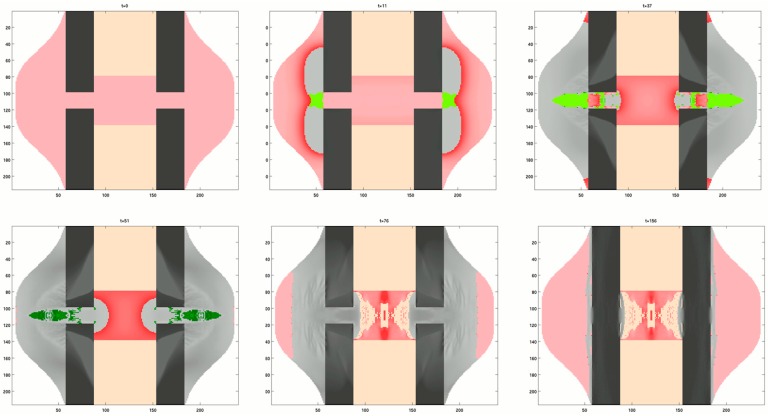
Six snapshots of a computer model showing the time evolution of different tissues during bone healing. The succession of images should be read from left to right starting with the top row. A top/bottom and left/right symmetry is assumed in the model. The starting configuration shows the disconnected cortical bone (black) surrounded by a callus of soft tissue (red) and bone marrow (orange). During the course of healing cartilage is formed (green) within the callus. Darker shades of the same color denote more mature tissue. In the case of bone, darker grey refers to a bone of higher mass density.

In the case of bone healing, the control function coupled the mechanical stimulus with the formed tissue type. Bone does not only have a much higher stiffness than cartilage (approximately, $E_{cart}$ = 500 MPa and $E_{bone}$ = 20 GPa), but the stiffening rate is much higher for bone compared to cartilage. The control function in bone healing is such that a low mechanical stimulation results in bone formation and therefore a rapid increase in stiffness, while a large mechanical stimulus leads to cartilage formation, i.e., a slow stiffening. The result of this rather counterintuitive coupling between stimulation and material healing response can be seen in Figure 4. Bone formation starts at the outer (i.e., periosteal) surface rather close to the fracture gap. The first bridging by newly formed tissue occurs via cartilage. Ossification includes the substitution of cartilage by bone. Finally, the superfluous bone is resorbed, while the density of the bone in the fracture gap attains the values of cortical bone.

Using mechanobiological models of bone healing, we studied the influence of (i) the size of the fracture gap [58,59], (ii) the different loading conditions including the elongation of the long bone by distraction osteogenesis [60], (iii) the stochasticity in the cellular mechanosensitivity [61], and (iv) the animal species [62] on the course of healing.

#### 3.3.2. Generic Model of Self-Repair in a Mechanoresponsive Material

**Figure 5:**
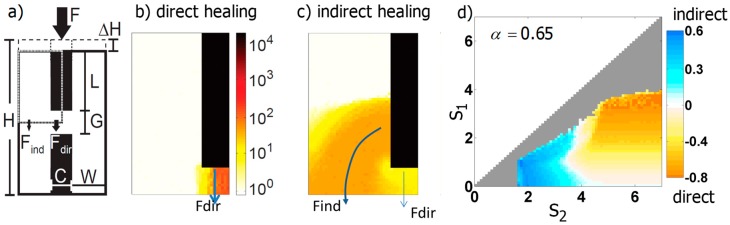
(**a**) 2D geometry of the model, where the black rectangles correspond to the disconnected stiff material surrounded by a soft, mechanoresponsive material (white). Loading of the broken material by a force *F* results in a vertical deformation Δ*H*. Due to symmetry, only a quarter of the system shown had to be modeled (marked by the dashed line). (**b**,**c**) Snapshots of two different simulations, which demonstrate that healing can progress either directly via a bridging of the fracture gap or indirectly, by reconnecting the broken ends circumferentially. (**d**) Parameter study varying the upper and lower bound of the range of mechanoresponsiveness, *s*_2_, and *s*_1_, respectively [63]. The colors denote whether the course of healing was more direct or indirect. Grey pixels indicate an unsuccessful healing defined by a failed reduction of Δ*H* below 1%.

The scenario of a stiff fractured material that is embedded in a mechanoresponsive material and heals under mechanical stimulation was systematically investigated in [63] (Figure 5a). The healing response consists in a local increase of the relative stiffness of the material, $\frac{\Delta E\left(x,t\right)}{E\left(x,t\right)},$ by a fixed amount, in case the local mechanical stimulus is within a range defined by the upper and lower bound, $s_{1}$ and $s_{2}$, respectively:

(3) $$\frac{\Delta E\left(x,t\right)}{E\left(x,t\right)}=\{\begin{array}{cc} C\;\Delta t & \mathrm{if}\;s_{1}\leq \left|\varepsilon \left(x,t\right)\right|\Delta {\left(\frac{E\left(x,t\right)}{E_{0}}\right)}^{\alpha }\leq s_{2}\\ 0 & \mathrm{otherwise} \end{array}$$

where |ε(x,t)| denotes the norm of the strain along the vertical loading direction (Figure 5a), $E_{0}$ the initial stiffness of the mechanoresponsive material, Δt the time increment, and C a proportionality factor. Since the mechanoresponsive material was assumed linear, the mechanical stimulus, $\left|\varepsilon \left(x,t\right)\right|\Delta {\left(\frac{E\left(x,t\right)}{E_{0}}\right)}^{\alpha }$, can be tuned from strain-like to stress-like by changing the exponent α from equal to 0 to a value of 1. In the simulations, all three control parameters of the mechanoregulation, $s_{1},$ $s_{2}$, and α varied systematically. The simulations showed very distinct healing courses, where healing occurred either by a stiffening of the material within the fracture gap and therefore a direct reconnection of the two broken pieces (direct healing, Figure 5b) or by a bridging of the broken pieces via material stiffening outside of the fracture gap (indirect healing, Figure 5c). The distinction between direct and indirect healing was evaluated by the ratio of force flowing via the fracture gap and outside of the fracture gap (Figure 5b,c).

The simulations demonstrated that the course of healing can be easily manipulated by the range of mechanical stimuli, in which the material shows a healing response. For an intermediate choice of the exponent (α=0.65), indirect healing outcomes were obtained when the range of mechanoresponse was rather limited or had a lower upper bound [63] (Figure 5d).

## 4. Conclusions and Implications for Synthetic Self-Healing Materials

The examples of bone remodeling and healing may provide inspiration for applications in very different scenarios. The local replacement of damaged material by remodeling is a typical example of biological damage management, which does not only extend the life time of the material, but also reduces the effort related to monitoring the damage. Remodeling also enables mechanical adaptation to applied loads. In contrast, bone healing is more complex as it has to initiate a process joining the broken pieces. An interesting aspect in healing is the strategy that the fracture callus providing the initial attachment is transiently far from an optimal repair. Only after a reconnection happened through the formation of a hard callus, a mechanical adaptation process sets in. The tissue then transforms itself into bone of the “correct geometry” so that the outcome of healing is a full return to the original unfractured state.

A key message from this perspective is the very different mechanoregulation in remodeling and healing. In bone remodeling, the material stays in a homeostatic state, where the control rule that relates mechanical stimulation with a change in material properties is characterized by a positive slope. This means that a larger mechanical stimulation leads to an effective stiffening, which in turn reduces the mechanical stimulation. Stimulation and material response are organized in a negative feedback loop (Figure 1), which maintains the bone structure in a dynamic equilibrium. For bone healing, the regulation is exactly opposite. During the formation of the fracture callus, stiff bone grows preferentially in sites of small mechanical stimulation, while much softer cartilage is formed in mechanically strongly stimulated regions. Consequently, the control rule is characterized by a negative slope between stimulation and stiffening and mechanoregulation can be described by a positive feedback loop (Figure 1). This would theoretically lead to an unlimited growth of bone mass, an escalation that is the fingerprint of positive feedback. However, this is not the endpoint of the healing process, since remodeling controlled by a negative feedback loop sets in at later stages and reduces the hard callus reverting the structure to the original one before fracture. The overshooting of the healing process by producing much more bone than it seems necessary can be interpreted as a transition between two different aims. The first aim is to stabilize the fracture, thus avoiding large movements of the fracture ends and leading to a bony reconnection. Later, after the mechanical stabilization occurred, the adaptation process leads the structure back into its homeostatic state.

The examples of different models explored in the context of repair mechanisms in bone were chosen to demonstrate the variety of control functions that were considered in different models. The slope of the control function can be positive or negative, it can be linear or non-linear (e.g., power law, sigmoidal function), and can include thresholds of the mechanical stimulation. In addition, different assumptions are often made about how the material responds locally to mechanical stimulation: if the material can undergo structural changes by addition or removal of material, it can increase or decrease its stiffness or it can change the local amount of damage. Similar damage models as presented here for bone are used, such as in the context of self-healing cementitious materials [64].

Finally, the mechanoregulation of adaptive responses in synthetic materials within the framework of the control function is introduced above. Specifically, we discussed two material classes with mechanoresponsive building blocks with very different sizes: mechanical metamaterials and polymers including mechanophores.

**Figure 6:**
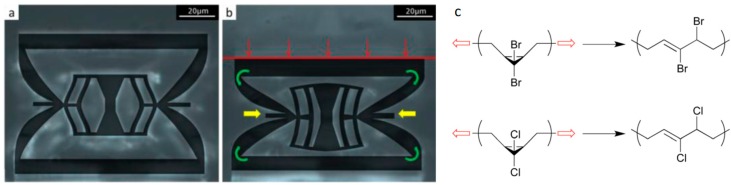
(**a**) Basic polymeric building block of a programmable metamaterial obtained by 3D printing in its initial state. (**b**) The intricate structure of this building block implies that under uniaxially loading (red arrows) above a specific threshold the bistable element in the center of the building block changes to a different configuration by snapping through. This structural change is accompanied by a change of the mechanical properties (from [70] with permission). (**c**) Two examples of molecules including mechanophores. Both belong to the class of gem-dihalocyclopropanes (top: gem-dibromocyclopropane, bottom: gem-dichlorocyclopropane). When applying a force as indicated by the red arrows, both molecules undergo a disrotatory ring opening reaction resulting also in a change of their mechanical properties. Using single molecule force spectroscopy experiments, the necessary force to trigger the ring opening reaction were determined to be 1210 pN and 1330 pN, respectively (from [71] licensed under CC BY 3.0). In contrast to the control functions described for processes in bone, both the mechanical metamaterial and the polymer including a mechanophore are characterized by a control function with an abrupt change in the mechanical properties at the setpoint of the mechanical stimulus.

The idea behind metamaterials is the use of a designed arrangement of tailored building blocks to obtain a material with unexpected effective properties. In an inverse design process, the desired material properties are pre-defined and the adequate microstructure is then searched for [65,66]. With this approach, a cubic metamaterial was produced that, under uniaxial loading, can display specific patterns on the surface that are created by protrusion [67]. In our context, an important subgroup of metamaterials are so-called programmable materials [68,69]. A recent example of a programmable metamaterial is based on a polymeric building block including a mechanically bistable element (Figure 6a). Loading the building block uniaxially above a specific threshold load triggers the bistable element to change to a second configuration by snapping through (Figure 6b). This structural change is accompanied by a change of the mechanical properties. Unloading the building block results in a return to the original state on the long run due to stress relaxation in the polymer [70]. The corresponding control function of the building block that connects uniaxial strain and stiffness is described by a step-function denoting a jump between the two states. Such a step-like behavior can be easily mapped on a conditional statement of a “if … then … else” form, known from computer programing. The structural state—in one of two possible states offered by the bistability—can be described as a material memory element reset after some time. The analogy with computer programs suggests that the question of whether the development of programmable materials means to improve the material “hardware” or more its “software”. In the example described above, the building block with the bistable element seems to correspond to a hardware component. The function of this hardware element could be manipulated, for example, by changing the setpoints in the “if … then … else” condition by applying external loads or other external force fields. In the case that many mechanoresponsive building blocks are incorporated into a material, the material between the building blocks deserves attention. This material provides coupling between the building blocks and is crucial for a possible collective behavior of the building blocks. The properties of this coupling element define the information transferred between building blocks. For example, if mechanical stress is the relevant stimulus, the coupling element can be designed to transfer only shear or only normal stresses. Furthermore, signals between building blocks can be dampened or strengthened by using lever elements. Again, by changing external loading conditions these coupling elements could be manipulated to transfer stresses and strains differently and change the overall response to external loads. While the hardware in terms of building blocks and coupling elements is fixed, the external loading and manipulations change the local processing and therefore could be intuitively considered as a software running on the hardware.

However, we think that a clear-cut distinction between hardware and software is not the appropriate view on programmable materials. Preferable seems a viewpoint that considers different layers of control starting from more basic layers to top layers responsible for the fine-tuning of material behavior. A possible feedback between the different control layers does not allow to describe these control layers in the form of a hierarchical stacking. In returning to the biological example of bone remodeling, the most basic layer would be the control rule for bone formation and resorption, as shown in Figure 2. Systemic changes in the body due to hormones would correspond to a control layer, which is thought to change setpoints in the control rule [30]. In addition to this endocrine signaling, a cell-to-cell communication via paracrine signaling could further fine-tune the mechanobiological control of bone remodeling.

Polymeric materials with mechanophores are a second example in which mechanical stimulation results not only in structural changes but in a change of mechanical properties. Mechanophores are force reactive groups that are embedded along the polymer backbone or within cross-links. The function of the mechanophore is that a local overloading does not result in molecular scission events, but in a constructive response with a local strengthening of the material [71] (Figure 6c). On the molecular level, the response can be a ring opening in the polymer, which provides not only stress relieve but can also trigger the generation of new cross-links [72]. A big challenge for the practical use of mechanophores is that levels of activation are very low (typically below 1%), even when the overall deformation is large and irreversible. On a molecular level, we encounter again an approximately step-like control function, where the molecular configuration of the mechanophore and with it the mechanical properties of the polymer change substantially at a certain threshold value of the applied force (often in the range of 1000 pN, see also the examples in Figure 6c) [71]. This setpoint can be manipulated by introducing the mechanophore in different polymer backbones. The force transduction through the polymer handle can result in a lever-arm effect causing a lowering of the setpoint force [73]. The use of localized mechanical forces to trigger chemical reactions was recently used to design a double-network hydrogel that adapts to mechanical loading. While the function of one network is to initiate polymerization reactions, which strengthen the network by “growing”, the other network avoids the problem of irreversible deformation by conserving the shape of the material. Similar to structural adaptation in muscles and bones, the material response occurs only at regions with a sufficient mechanical stimulus [74].
